# Water structure changes in oxime-mediated reactivation process of phosphorylated human acetylcholinesterase

**DOI:** 10.1042/BSR20180609

**Published:** 2018-06-29

**Authors:** Irina V. Zueva, Sofya V. Lushchekina, Patrick Masson

**Affiliations:** 1 A.E. Arbuzov Institute of Organic and Physical Chemistry of Russian Academy of Sciences, Arbuzov str. 8, Kazan 420088, Russia; 2 N.M. Emanuel Institute of Biochemical Physics of Russian Academy of Sciences, Kosygina str. 4, Moscow 119334, Russia; 3 Kazan Federal University, Pharmacology Laboratory, Kremlevskaya str, 18, Kazan 420008, Russia

**Keywords:** acetylcholinesterase, lyotropic salts, molecular dynamics, organophosphate, oxime, reactivation

## Abstract

The role of water in oxime-mediated reactivation of phosphylated cholinesterases (ChEs) has been asked with recurrence. To investigate oximate water structure changes in this reaction, reactivation of paraoxon-inhibited human acetylcholinesterase (AChE) was performed by the oxime asoxime (HI-6) at different pH in the presence and absence of lyotropic salts: a neutral salt (NaCl), a strong chaotropic salt (LiSCN) and strong kosmotropic salts (ammonium sulphate and phosphate HPO_4_^2−^). At the same time, molecular dynamic (MD) simulations of enzyme reactivation under the same conditions were performed over 100 ns. Reactivation kinetics showed that the low concentration of chaotropic salt up to 75 mM increased the percentage of reactivation of diethylphosphorylated AChE whereas kosmotropic salts lead only to a small decrease in reactivation. This indicates that water-breaker salt induces destructuration of water molecules that are electrostricted around oximate ions. Desolvation of oximate favors nucleophilic attack on the phosphorus atom. Effects observed at high salt concentrations (>100 mM) result either from salting-out of the enzyme by kosmotropic salts (phosphate and ammonium sulphate) or denaturing action of chaotropic LiSCN. MDs simulations of diethylphosphorylated hAChE complex with HI-6 over 100 ns were performed in the presence of 100 mM (NH_4_)_2_SO_4_ and 50 mM LiSCN. In the presence of LiSCN, it was found that protein and water have a higher mobility, i.e. water is less organized, compared with the ammonium sulphate system. LiSCN favors protein solvation (hydrophobic hydration) and breakage of elelectrostricted water molecules around of oximate ion. As a result, more free water molecules participated to reaction steps accompanying oxime-mediated dephosphorylation.

## Introduction

Cholinesterases (ChEs) are irreversibly inhibited by organophosphorus esters (OPs) through phosphorylation of their active site serine [1].Owing to the physiological function of acetylcholinesterase (AChE, EC 3.1.1.7) in the cholinergic system in terminating the action of the neurotransmitter acetylcholine, acute poisoning by OPs causes a major cholinergic syndrome [2]. The emergency therapy of OP poisoning is based on the reactivation of phosphylated AChEs by strong nucleophilic compounds, i.e. oximes (for review, see [3], an antimuscarinic (atropine) and an anticonvulsant (benzodiazepine)). Bioscavengers that neutralize OP molecules in the bloodstream can be administered with benefits in persistent systemic exposure [2,4]. Oxime reactivators of ChEs and bioscavengers can be regarded as antidotes. However, there is no universal reactivator. Failure of oximes to fully reactivate all the phosphylated ChEs may results from (i) sterical and/or chemical (electronic) reasons dependent on both the structure of the phosphyl adduct, the structure of reactivator, and its binding in the enzyme active center [5,6]; (ii) a post-inhibitory process called ‘ageing’, dealkylation of an alkoxy chain on the OP adduct, making the aged adduct refractory to oxime-mediated reactivation [7,8]. Moreover, in some cases, reactivated AChE can be subsequently reinhibited by phosphylated oxime that can be more potent than the initial OPs [9]. Thus, for a better understanding of the reactivation process, it is mandatory to improve the medical countermeasures against OP poisoning.

The reactivation reaction – displacement of the phosphyl adduct by a nucleophile, i.e. oximate ion – implies (i) structural/sterical requirements (structure of the reactivator allowing good positioning and optimal distance of the oxime function in the enzyme active site gorge for nucleophilic attack of the P atom); (ii) chemical requirements (p*K*_a_∼7–8 of oximate); partial positive charges on the P atom; dielectric constant not too low at the bottom of the active site gorge compared with that of water); (iii) environmental requirements: pH of the medium, water molecules in the active site gorge, presence or not of co-solvent, temperature, pressure (hydrostatic, osmotic), medium viscosity). If the ageing reaction is slow compared with the reactivation, the percentage of reactivation of phosphylated enzyme depends on these factors.

**Scheme 1:**
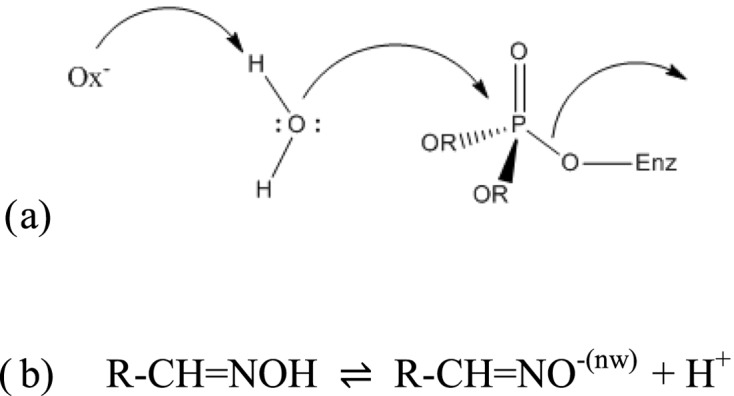
Oxime-mediated reactivation of phosphorylated ChE (**a**) Direct role of water in reactivation; (**b**) oxime-oximate equilibrium showing water molecules electrostricted around oximate function

Numerous works showed that the pH dependence of oxime-mediated reactivation of phosphylated ChEs is described by a bell-shaped curve [10,11]. This curve is similar to the enzyme activity pH dependence, assuming that reactivation is controlled by protonated histidine (His^447^) of the phosphylated catalytic triad (p*K*_a_ = 7.3), glutamate (Glu^202^) adjacent to the catalytic serine (Ser^203^), and the oximate form of reactivator (p*K*_a_ = 7–8). Recent QM/MM works showed that His^447^ and Glu^202^ work together to deprotonate the oxime reactivator [12]. However, interactions between sterical, chemical, and environmental factors may alter both pH dependence and yield of reactivation. In particular, it was hypothesized that the water may act in reactivation process, either as direct nucleophile after being polarized by oximate (Scheme 1a) [13] or that solvation/electrostriction of water molecules (nw) around the negative oximate oxygen (Scheme 1b) exerts a pronounced shielding effect as the pH increases beyond pH > p*K*_oxime_, which in turn would lower the nucleophilicity of the oximate anion. In the latter case, nucleophilic action of oximate implies desolvation prior attack.

The active site gorge of ChE is filled with water molecules, some of them are free, others are cluster organized [14]. Water acts as a co-substrate in hydrolytic activity. In addition, the importance of water in ageing of phosphylated ChE [15,16], ligand binding, conformational competence, and molecular dynamics (MD) [14,17,18] has been recognized.

Thus, to investigate the effect of solvation/electrostriction on dephosphorylation of diethylphosphate hAChE, reactivation of paraoxon-inhibited AChE was performed using the oxime asoxime (HI-6) compared with pH in the presence of lyotropic salts (Hofmeister series of salts): a chaotropic salt, i.e. a water structure breaker (lithium thiocyanate, LiSCN), kosmotropic salts, i.e. water structure makers (ammonium sulphate, sodium phosphate), and a lyotropically neutral salt (NaCl) [19]. Results showed that in the presence of a chaotropic salt, the shielding effect of electrostricted water molecules was decreased, and then reactivation yield was slightly increased. In the presence of non-lyotropic salts (middle of the Hofmeister series) there was no effect. In the presence of kosmotropic salts (water structure makers) the shielding effect was slightly strengthened and reactivation yield decreased.

MD simulation on complex of diethylphosphorylated enzyme with HI-6 in the presence of kosmotropic and chaotropic salts over 100 ns support these observations and indicate a better availability of free water molecules in the presence of LiSCN, improving nucleophilic displacement of the phosphoryl adduct.

## Material and methods

### Enzyme and chemicals

Highly purified recombinant human AChE was expressed in CHO (Chinese-hamster ovary cells [6]. Echothiophate was a gift from Biobasal (Basel, Switzerland). Paraoxon was from Sigma–Aldrich. HI-6 dihydrochloride was a gift from Pharmsynthez (Saint Petersburg, Russia). All other chemicals of biochemical grade were purchased from Sigma–Aldrich.

### Inhibition of hAChE

hAChE was titrated by echothiophate iodide as described [20]. Ninety five percent inhibition of hAChE (2 × 10^−9^ M) by paraoxon (8 × 10^−8^ M) was performed in 10 mM Bis/Tris propane/HCl buffer at pH 7.0 for 2.5 h at 25°C.

### Reactivation of diethylphosphorylated hAChE

Diethylphosphorylated AChE after inhibition by paraoxon was reactivated by the oxime HI-6 (p*K*_a_ = 7.63 [21], = 7.47 [22], = 7.13 [23]). Inhibited AChE was immediately incubated at 25°C with 1 mM HI-6 in 10 mM Bis/Tris propane buffer in the absence and presence of salts, sodium chloride and lyotropic salts, lithium thiocyanate, ammonium sulphate, and sodium phosphate. NaCl was used as neutral lyotropic salt; LiSCN as strong chaotropic salt, and (NH_4_)_2_SO_4_ as a strong kosmotropic salt. In addition, other reactivation experiments were performed in sodium phosphate buffer of different molarities because of the kosmotropic properties of phosphate ions. Most salt concentrations were 50, 100, 250, 500, 1000 mM, except for LiSCN where a wide range of concentrations lower than 100 mM were investigated at pH = 8. The pH of buffers ranged from 6.5 to 9.5; the pH interval between the different buffers was 0.5 unit. Under each buffer condition, the time-dependent percentage of reactivation of AChE (A_t_) was calculated using eqn 1 [24,25]:

(1) $${\text{A}}_{\text{t}}={\text{A}}_{0}(1-\exp(-{\text{k}}_{\text{obs}}.t))$$

where A_0_ is the control activity of non-inhibited enzyme and *k*_obs_ is the apparent first order reactivation constant with 1 mM HI-6.

Activity measurements of reactivated enzyme were performed at 25°C using the Ellman method [26] in 0.1 M sodium phosphate buffer pH 8.0 with 1 mM acetylthiocholine (ATC) as the substrate after 5, 10, 15, 20, 30, 40, 60 min incubation with HI-6 until the plateau for maximum of reactivation (A_∞_) was reached. Spontaneous hydrolysis of ATC due to the presence of HI-6 was subtracted.

### Specific effect of LiSCN on AChE activity at pH 8.0

Because chaotropic salts are known to affect the conformational stability of proteins, inducing unfolding activity measurements of non-inhibited AChE were performed at varying concentrations of LiSCN, up to 1M, at pH 8.0. This allowed the determination of LiSCN-induced enzyme inactivation that precedes overall enzyme unfolding. To eliminate possible artefactual results due to the nucleophilic properties of SCN^−^, the action of LiSCN on dithiobis-nitrobenzoic acid (DTNB), the chromogenic reagent of Ellman assay (final concentration of DTNB was 0.1 mM), was checked.

### MD

Two model systems were set up: hAChE/HI-6/Bis/Tris propane/100 mM (NH_4_)_2_SO_4_ and hAChE/HI-6/Bis/Tris propane/50 mM LiSCN.

The X-ray structure of human AChE *apo*-state was from PDB ID: 4YE4 [27]. Its catalytic serine was diethylphosphorylated. Then, the position of HI-6 in the gorge (deprotonated form) was taken from PDB ID: 2WHP for mouse AChE whose 3D structure is similar to that of human enzyme [21]. TIP3P water molecules were added to form 170 Å × 170 Å × 170 Å rectangular water box. Number of other molecules were added with respect to their concentrations in the experimental system at pH 8.0, keeping in mind the problem of use of macroscopic parameters, like pH and molar concentrations, for such a small cell (≈5000 nm^3^) and classical MD simulations: thus, two HI-6 hydrochloride molecules outside the protein were added to the one located inside hAChE (two deprotonated oximate molecules and one protonated); 31 Bis/Tris propane molecules (p*K*_a1_ = 6.8, p*K*_a2_ = 9.0 (https://www.sigmaaldrich.com): 3 neutral, 26 monoprotonated, 2 biprotonated forms); 300 SO_4_^2−^ and 600 NH_4_^+^ ions for 100 mM (NH_4_)_2_SO_4_ system and 150 Li^+^ and 150 SCN^−^ for the system with 50 mM LiSCN. Bis/Tris propane molecules and ions were added as regular grids with help of VegaZZ software [28]. Total number of atoms were close to 500000.

Parameters for organic molecules and ions were taken from the Charmm General Force Field [29–31] with help of CGenFF interface (https://cgenff.paramchem.org), certain parameters, which were not available there, were parameterized with help of *ff*TK plugin of VMD [32,33].

Because ions and molecules were added to the solution as regular grids, before productive runs, systems were pre-equilibrated with protein atoms fixed during 10 ns at 363 K, and then gradually cooled down to 298 K during 1 ns. Distribution of molecules and ions in the system was controlled with the help of radial distribution function calculations [34]. After pre-equilibration of the solvent, the systems were fully minimized during 2000 steps, and productive 100 ns MD runs were performed in NPT ensemble, 298 K, 1 atm, periodical boundary conditions.

For all MD simulations NAMD 2.11 software [35] with CHARMM36 force field [36] was used. MD simulations were run at the Lomonosov Moscow State University supercomputer [37]. For the preparation of the model systems and further analysis of MD trajectories VMD [38] and ProDy software [39] were used. Figures were prepared with VMD and PyMol software (Schrödinger LLC; http://www.pymol.org).

## Results and discussion

### pH dependence of reactivation in the presence of varying concentrations of lyotropic salts

**Figure 1:**
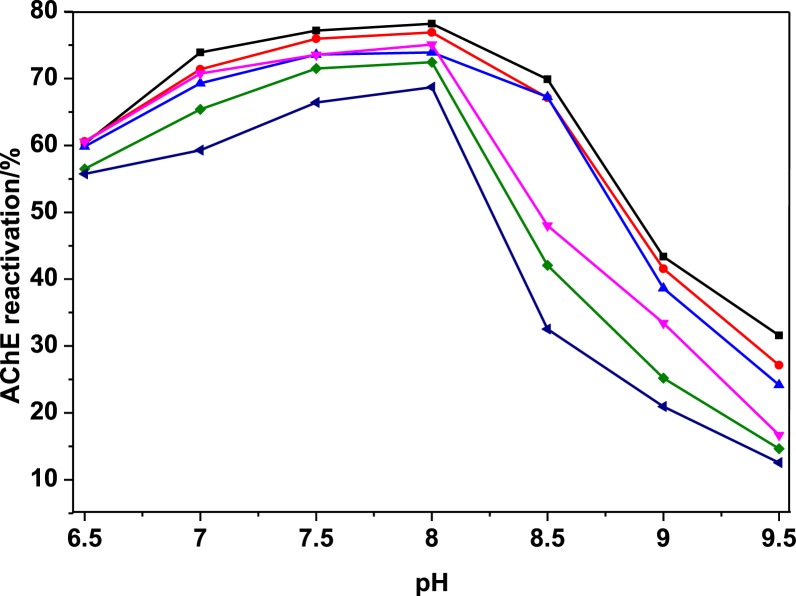
pH dependence of HI-6-mediated reactivation of diethylphosphorylated human AChE in the presence of ammonium sulphate, a strong kosmotropic salt ▪, no salt addition; (NH_4_)_2_SO_4_ added to buffers: 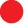, 50 mM; 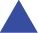, 100 mM; 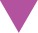, 250 mM; 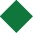, 500 mM; 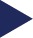 1000 mM.

At pH = 8, the optimum pH, 1 mM HI-6 is capable of reactivating 77.5 ± 1.1% of diethylphosphorylated human AChE. This is in agreement with values reported by several investigators, e.g. 92% in 1 h with 1 mM HI-6 at 37°C [40]. The presence of lyotropic salts did not alter the pH profile of reactivation. This indicates that the status of water in the active site gorge, altered by salts, has no effect on the protonation state of residues involved in the reactivation mechanism. The reactivation rate decreased slowly as salt concentration increased for the lyotropically neutral salt NaCl, suggesting that decrease resulted from change in viscosity of the medium and possible non-specific ionic strength effect on electrostatic interactions. In the case of phosphate, strong decrease in reactivation was observed. This may reflect the strong kosmotropic effect of phosphate (HPO_4_^2−^ is the major species at pH = 8) that may quench the nulcleophilicity of the oximate. However, a direct interaction between phosphate ions and positively charged oxime cannot be ruled out, decreasing the effective oxime concentration, and therefore the maximum of reaction. With sulphate ions, a moderate decrease in reactivation was also observed at all pHs as salt concentration increased (Figure 1). This may also reflect the kosmotropic effect of sulphate on electrostriction of water molecules around the oximate ion.

**Figure 2:**
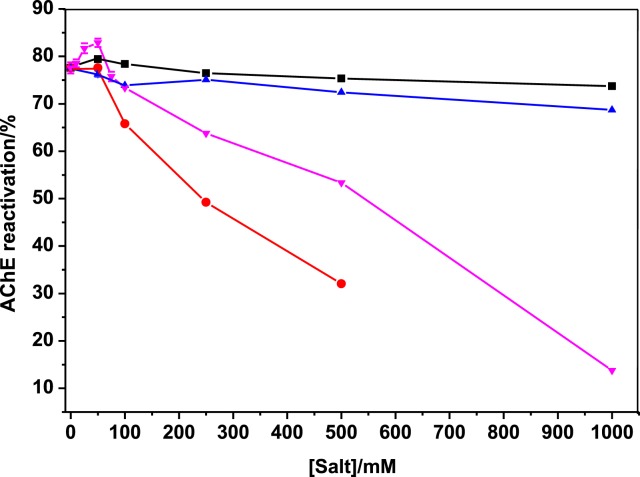
Lyotropic effects of Hofmeister series salts at optimum pH of reactivation (pH = 8) ▪, NaCl; 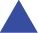, (NH4)_2_SO_4_; 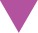, LiSCN; 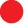, sodium phosphate buffer.

Unlike the other salts, the strong chaotropic salt lithium thiocyanate showed a clear activating effect at concentrations lower than 100 mM, in particular at optimum pH, with a maximum yield of 82.9 ± 0.9% for 50 mM LiSCN (Figure 2). LiSCN as a water-structure breaker induces destructuration of water molecules electrostricted around oximate. Thus, desolvation of oximate favors nucleophilic attack on the phosphorus atom.

Above 100 mM reactivation yield dropped. Because LiSCN is a strong chaotropic salt, acting as water-breaker, the decrease in reactivation above 100 mM cannot result from a shielding effect of water molecules around the oximate function.

**Figure 3:**
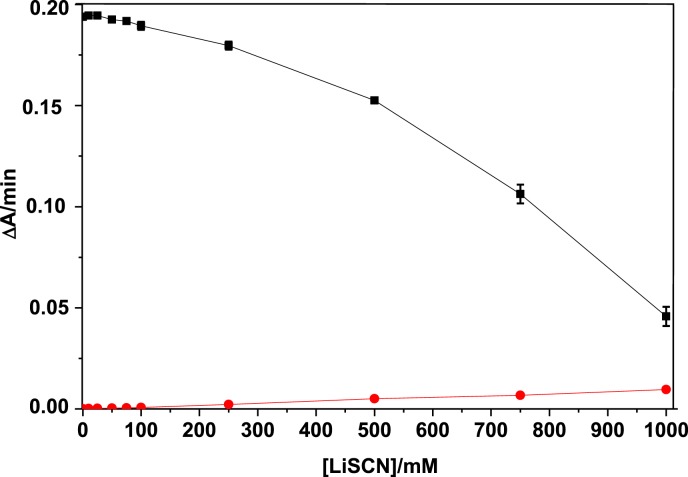
Effect of LiSCN on AChE activity (▪) with 1 mM ATC at pH 8 The red curve shows the weak reducing action of SCN^−^ on DTNB.

Although thiocyanate displayed nucleophilic properties (in (S=C=N)^−^, the charge was equally distributed between N and S atoms), it did not significantly reduce DTNB. Thus, SCN^−^ did not interfere with AChE activity measurements (Figure 3). The effect of LiSCN on non-inhibited AChE showed a decrease in enzyme catalytic activity, resulting from denaturation (denaturation mid-point is close to 800 mM). Moreover, the catalytic activity was not increased at low LiSCN concentrations (in the absence of LiSCN, activity was 0.193 ΔA/min, in the presence of 50 mM LiSCN was 0.192 ΔA/min) (Figure 3). This supports the conclusion that increase in reactivation at low LiSCN did not result from increase in ionic strength but resulted from a lyotropic effect.

In other words, the effects observed at high salt concentrations (>100 mM) result either from salting-out of the enzyme by kosmotropic salts (phosphate and ammonium sulphate) or denaturing action of chaotropic LiSCN [41,42].

Therefore, the significant increase (7%) in HI-6-mediated reactivation of AChE in the presence of 50 mM LiSCN reflects the increase in nucleophilicity of the oximate ion due to destructuration of electrostricted water molecules around the oximate ion. These results fit with former reports. Indeed, entropy–enthalpy compensation phenomenon observed in reactivation of phosphorylated butyrylcholinesterase suggested the displacement of water molecules and conformational change during the oxime binding process of reactivation [43]. Acidity and kinetic measurements of proton transfer in water and water-DMSO mixtures for oxime reactions also support the oxime desolvation hypothesis prior to nucleophilic attack on electrophilic phosphorus atoms [23,44,45].

### MD

**Figure 4:**
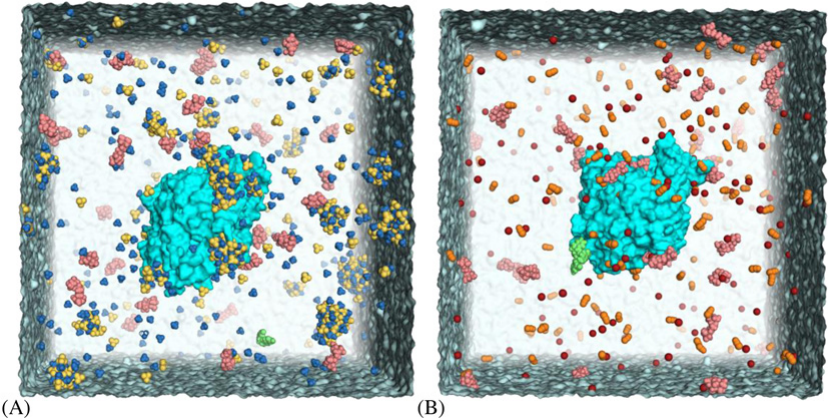
Systems for MD simulations Systems for MD simulations: hAChE with 1 mM HI-6 in 10 mM Bis/Tris propane and (**A**) 100 mM (NH_4_)_2_SO_4_ or (**B**) 50 mM LiSCN. Bis-tris propane molecules are colored pink, HI-6, green; NH_4_^+^, blue; SO_4_^2−^, yellow; Li, red; SCN^−^, orange.

**Figure 5:**
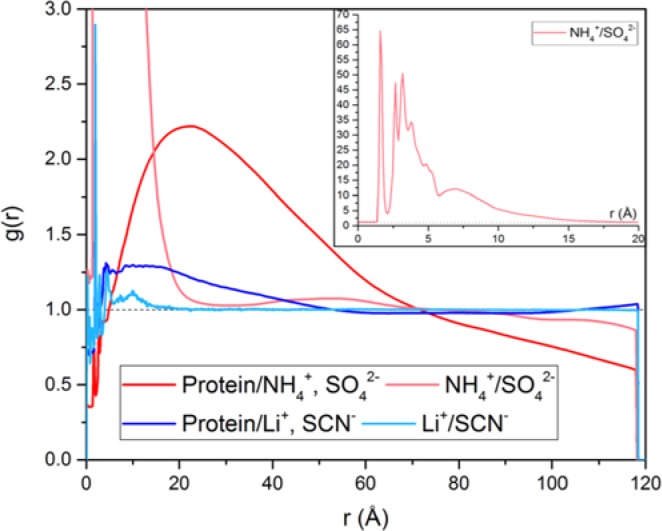
RDF of ions around protein and between counter-ions over 100 ns productive MD run; blue lines for hAChE/50 mM LiSCN system, and red lines for hAChE/100 mM (NH_4_)_2_SO_4_ system, inset shows RDF between NH_4_^+^ and SO_4_^2−^ at different scale, reflecting formation of aggregates Abbreviation: RDF, radial distribution function.

MD simulations were carried out in systems where optimal lyotropic effects on oxime reaction were observed: 100 mM (NH_4_)_2_SO_4_ and 50 mM LiSCN. During MD simulations (NH_4_)_2_SO_4_ small metastable aggregates were formed, some of them sticking to the protein surface (Figure 4). This illustrated kosmotropic nature of of (NH_4_)_2_SO_4_ in protein salting in at low concentration. Chaotropic ions of LiSCN were distributed evenly, with slightly increased density near the protein surface (Figures 4 and 5).

**Figure 6:**
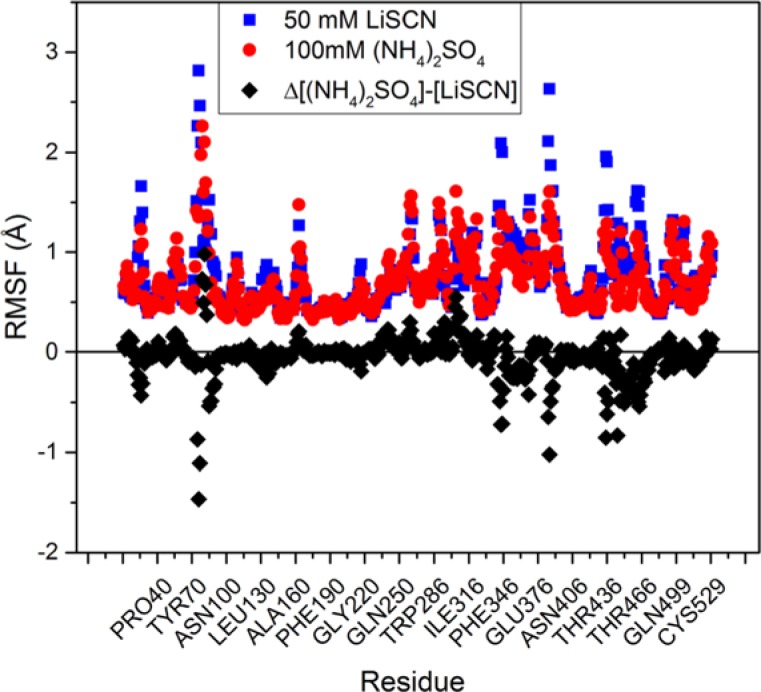
Per-residue RMSF over 100 ns trajectories for the two model systems, black points show RMSF difference (Δ) between hAChE/100 mM (NH_4_)_2_SO_4_ and hAChE/50 mM LiSCN systems

**Figure 7:**
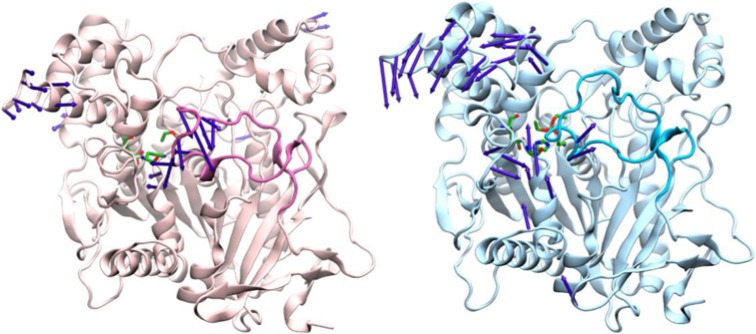
Dominant modes extracted from MD trajectories by PCA for hAChE/(NH_4_)_2_SO_4_ (hAChE is shown with pink ribbons) and hAChE/LiSCN (hAChE is shown with blue ribbons) systems

In the system containing (NH_4_)_2_SO_4_, the mobility of hAChE is decreased compared with the system containing LiSCN. This is reflected by root mean square fluctuations (RMSF) of the whole protein, excluding the most mobile N-terminal and C-terminal amino acids (residues 10–530 were included in RMSF calculations) along 100 ns MD trajectory was 1.2 ± 0.2 Å for hAChE/LiSCN system and 1.0 ± 0.1 Å for hAChE/(NH_4_)_2_SO_4_ system (RMSF calculation window 100 steps), means are significantly different at 0.05 level. Pre-residue RMSF are shown in Figure 6, in case of hAChE/(NH_4_)_2_SO_4_ system mobility is decreased for the most part of the protein, except for an upper part of the Ω-loop, the loop that links the peripheral anionic site to the catalytic binding site (Trp^86^). This fits with the results of principle component analysis (PCA) of MD trajectories, showing more mobile regions for hAChE in LiSCN solution, except for the upper part of the Ω-loop (Figure 7).

This influences hydration immediately around phosphorylated adduct: in hAChE/LiSCN system, isolated water molecules were found within 3.5 Å of phosphorus atom in 9.39% of snapshots, and only in 0.17% snapshots in the AChE/(NH_4_)_2_SO_4_ system. On the other hand, (NH_4_)_2_SO_4_ creates more stiff environment in the active site. This makes the presence of a free water molecule close enough to phosphorus more rare event. Higher number of water molecules in the vicinity of P atom due to higher overall protein and water molecules mobility (destructuration of the water molecule network electrostricted around oximate ion) in the presence of LiSCN facilitates desolvation of oximate-ion, and oxime-mediated reactivation. However, from these MD simulations we cannot decide how exactly isolated water molecules participate in the reactivation mechanism as depicted in Scheme 1a or in proton exchange between active site residues. Detailed mechanism could be revealed by constant pH MDs and QM/MM modeling [12].

**Figure 8:**
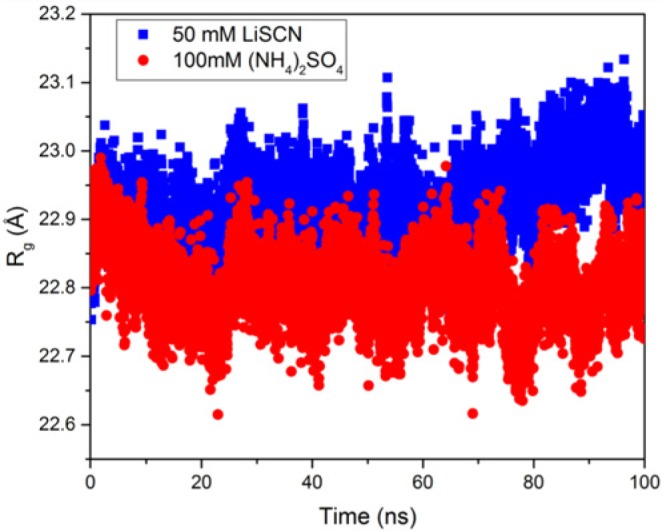
Radius of gyration of hAChE (C^α^ atoms) in the two model systems along MD trajectory

Increased mobility of protein and water molecules, thus increased hydrophobic hydration of the polypepdide chain in the presence of 50 mM LiSCN brings the enzyme close to the beginnig of denaturation (Figure 8). Increased *R*_g_ can reflect a transition toward a pre-denaturation molten globule state. Indeed, denaturation starts for >75 mM LiSCN (Figure 3, for free enzyme inactivation compared with LiSCN concentration).

## Conclusion

Present results showing an increase in oxime-mediated reactivatability of diethylphosphorylated human AChE at low concentration of a chaotropic salt support the hypothesis that desolvation of the oximate ion is needed prior to nucleophilic attack on the phosphorus atom. However, we cannot exclude that a polarized water molecule may participate in the nucleophilic attack on the phosphorus atom. Moreover, motion, dynamic structuration, and destructuration of water molecule clusters [12] in the active site gorge may affect the protonation pattern of important residues, Glu^202^ in particular, involved in the reactivation process, [12].

## Abbreviations

AChE: acetylcholinesterase
ATC: acetylthiocholine
ChE: cholinesterase
DTNB: dithiobis-nitrobenzoic acid
HI-6: asoxime
MD: molecular dynamics
OP: organophosphorus agent
PCA: principle component analysis
RMSF: root mean square fluctuation
